# Editorial for the Special Issue on Methodology, Microfabrication and Applications of Advanced Sensing and Smart Systems

**DOI:** 10.3390/mi15091149

**Published:** 2024-09-13

**Authors:** Luyu Jia, Shanling Ji, Yuze Gao, Haiying Wen, Jianxiong Zhu

**Affiliations:** 1 Sustainable Development Research Group, Chongqing University of Technology, Chongqing 400054, China; jialy@cqut.edu.cn; 2 Guangdong Provincial Key Laboratory of Electronic Functional Materials and Devices, Huizhou University, Huizhou 516001, China; 3 School of Mechanical Engineering, Southeast University, Nanjing 211189, China

Smart sensing and advanced systems have played crucial roles in the modern industrialization of society, which has led to many sensors being used in fabrication methodologies for various applications, such as in medical equipment [1], robotics activities [2], sustainable electronics systems [3], and smart devices with artificial intelligence and the Internet of Things (IoT) [4]. Whether combining machine learning technology with wearable sensors to monitor human activity [5,6] or applying sensors to a Triboelectric Nanogenerator (TENG) [7], these examples vividly demonstrate that sensing technology is increasingly deeply integrated with other cutting-edge technologies. Due to the requirements of these technologies, the methodologies used in the microfabrication of advanced sensing and smart systems have become increasingly sustainable and advanced [8], and the structural design is more ingenious, enhancing the sensor’s stability and responsiveness [9]. This Special Issue seeks to showcase the latest advancements in and contributions to the micro/nanofabrication, methodology, integration, and application of sensors and advanced systems with the aid of technologies in the fields of artificial intelligence, multisensor fusion, machine vision, human–machine interaction, machine learning, big data, advanced robotics, and others.

This Special Issue includes articles on intelligent manufacturing methodologies [10], fluorescence microscopy image inpainting [11], the Micro-Electro-Mechanical-System (MEMS) micromirror [12], wearable robotics in processing design [13], the quantitative analysis of particle size [14], and medical monitoring systems [15], which could give readers a glimpse of the challenges, opportunities, and development trends regarding machine learning in micro/nano sensing and systems design. To address the multivariable prediction of coated film and data augmentation problems during nanoscale coating manufacturing from big data, Ji et al. [10] design a novel auxiliary regression approach using a self-attention-augmented generative adversarial network (AR-SAGAN), which is verified to have lower thickness prediction errors than other traditional methods. In terms of image inpainting, in order to solve the problem of missing biological information due to saturation artifacts in fluorescence microscope images, Liu et al. [11] introduce the TC-GAN model, a two-stage phenotypic feature, which separately restores the shape and texture features of cells. The results demonstrate the model’s practical significance. To reduce the interference of vehicle vibration on MEMS micromirrors, Qian et al. [12] propose to implement a mechanical low pass filter (LPF) as a vibration isolator for a micromirror. The attenuation ratio is 0.51 in piston mode. The proposed design provides considerable attenuation for the micromirror, which relieves the stress on the slow flexure. To explore sustainable exoskeletons with efficient energy harvesting devices in design methodology, Shi et al. [13] propose the design of a lightweight wearable Bowden cable-actuated soft exoskeleton robot with energy harvesting capability, which shows an average of a 7.91% reduction in thigh muscle activity, with a maximum of 3.2 W of electric power being generated during movement downstairs. In terms of the quantitative analysis of particle size, Jiang et al. [14] establish a simulation model for a flavoring nozzle to investigate the atomization effect under different ejection pressures. The influencing factors of liquid particle size in two-phase nozzle flow are adopted to verify the cubic correction relationship between the simulation and experiment particle size. In terms of medical monitoring systems, Li et al. [15] focus on the successful modification of polyvinylidene fluoride (PVDF) by introducing polyvinyl alcohol, resulting in a novel piezoelectric polymer. This material demonstrates a more sensitive sensing ability in blood pressure monitoring systems, improving the accuracy of blood pressure monitoring.

We would like to thank all the authors and reviewers for their contributions to this Special Issue. We also hope that the articles showcased here are interesting and helpful for readers and inspire new innovations in the current state of the art regarding advanced sensing and smart systems.
